# Nail Discoloration Following the Chronic Use of Skin-Depigmenting Creams

**DOI:** 10.7759/cureus.67770

**Published:** 2024-08-25

**Authors:** Manuji Bandara, Achala Liyanage

**Affiliations:** 1 Internal Medicine, University of Ruhuna, Galle, LKA; 2 Dermatology, University of Ruhuna, Galle, LKA

**Keywords:** hydroquinone, skin depigmenting agent, skin lightening cream, nail hyperpigmentation, nail discoloration

## Abstract

Skin-depigmenting creams are on the rise, driven by societal pressures that equate fair skin with beauty, success, and happiness. This trend has led to many unregulated products being released on the market, causing various adverse effects on users. This case report examines five patients with nail hyperpigmentation following the use of skin-depigmenting creams containing hydroquinone (HQ). The findings from this case report showcase overlooked side effects of these widely used creams. Healthcare providers may be unfamiliar with the rare side effects of depigmenting creams, leading to misdiagnoses and unnecessary evaluations. Moreover, there is a pressing need for healthcare authorities to enhance regulation and for healthcare providers to be more vigilant in diagnosing presentations of nail hyperpigmentation. This case report provides valuable insights into the side effects of HQ-based creams as observed in the Asiatic population.

## Introduction

Skin hyperpigmentation significantly affects quality of life, bearing social, political, and cultural importance, especially among people with skin color [1]. Skin depigmenting is used to lower the concentration of melanin in hyperpigmented areas of skin, to achieve a fairer tone [1]. Chemical analysis of these products has identified harmful substances such as mercury, potent corticosteroids, and hydroquinone (HQ) [2-4]. These products often contain up to 10,000 ingredients, including high levels of toxic metals [3].

Consequently, these products are heavily marketed and widely available due to increased demand [3], resulting in the proliferation of unregulated online and over-the-counter (OTC) products. Complications of HQ that have been reported are dermatitis, exogenous ochronosis, cataracts, pigmented colloid milia, and scleral and nail pigmentation. A more serious complication from chronic use is loss of elasticity and impaired wound healing. Users who chronically bleach their skin may also exude an offensive fish odor in their sweat [5].

## Case presentation

Case 1

**Figure 1:**
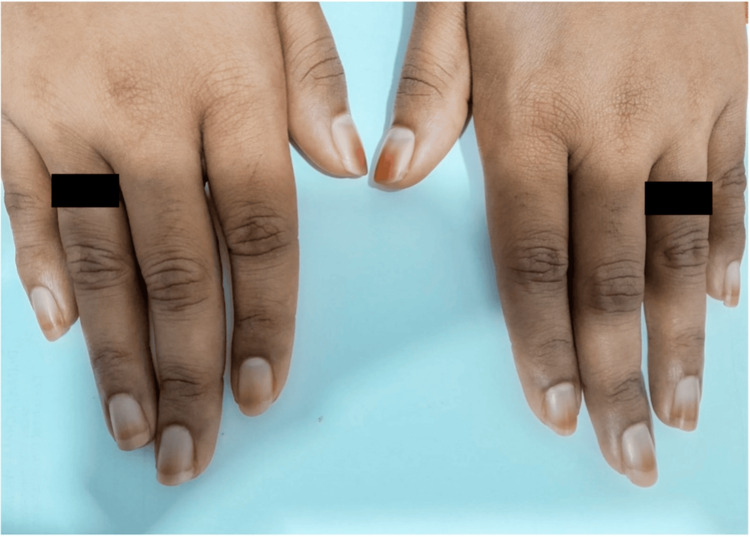
Clinical findings of Case 1

A 25-year-old female, with no prior co-morbidities, presented with hyperpigmentation of the nails, involving the distal nail plate and nail edges, approximately 4 mm from the proximal nail fold, which occurred following 14 weeks of use. The discoloration affected all fingernails on both hands with a more pronounced appearance on the bilateral first thumbnails (Figure 1). She had been using a skin-depigmenting cream over her face and hands for nearly six months, which she discontinued two months before the presentation.

Case 2

**Figure 2:**
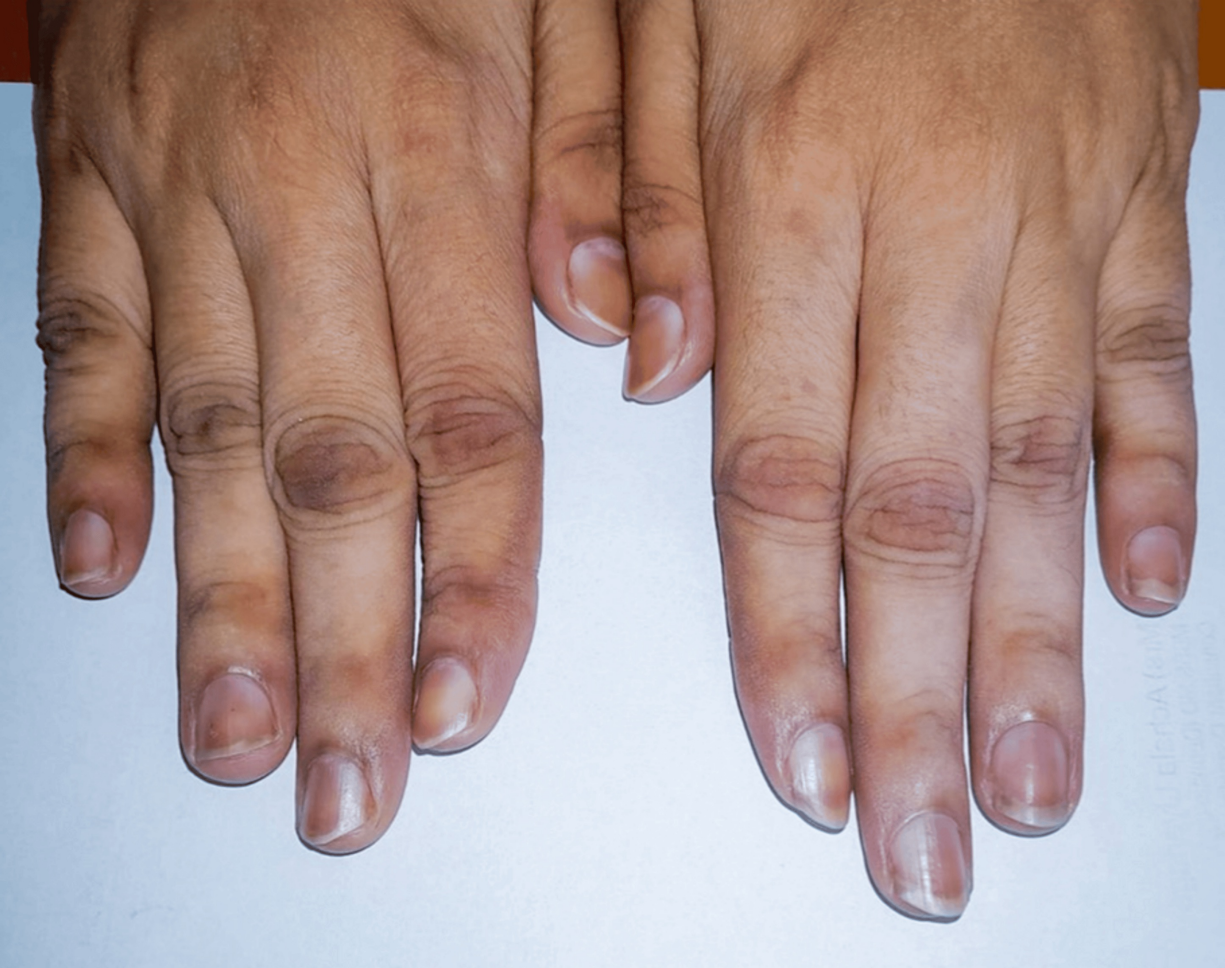
Clinical findings of Case 2

A 22-year-old previously healthy female presented with dusky orange hyperpigmentation of the nails following 15 weeks of use. The hyperpigmentation was located 2-3 mm from the proximal nail fold, as depicted in Figure 2. The pigmentation was in all the fingernails on both hands. Additionally, hypopigmentation was noted on the dorsum of her hands sparing the distal interphalangeal joints. She had used a skin-depigmenting cream over her face and hands for nearly five months, which she discontinued two months prior to presentation.

Case 3

**Figure 3:**
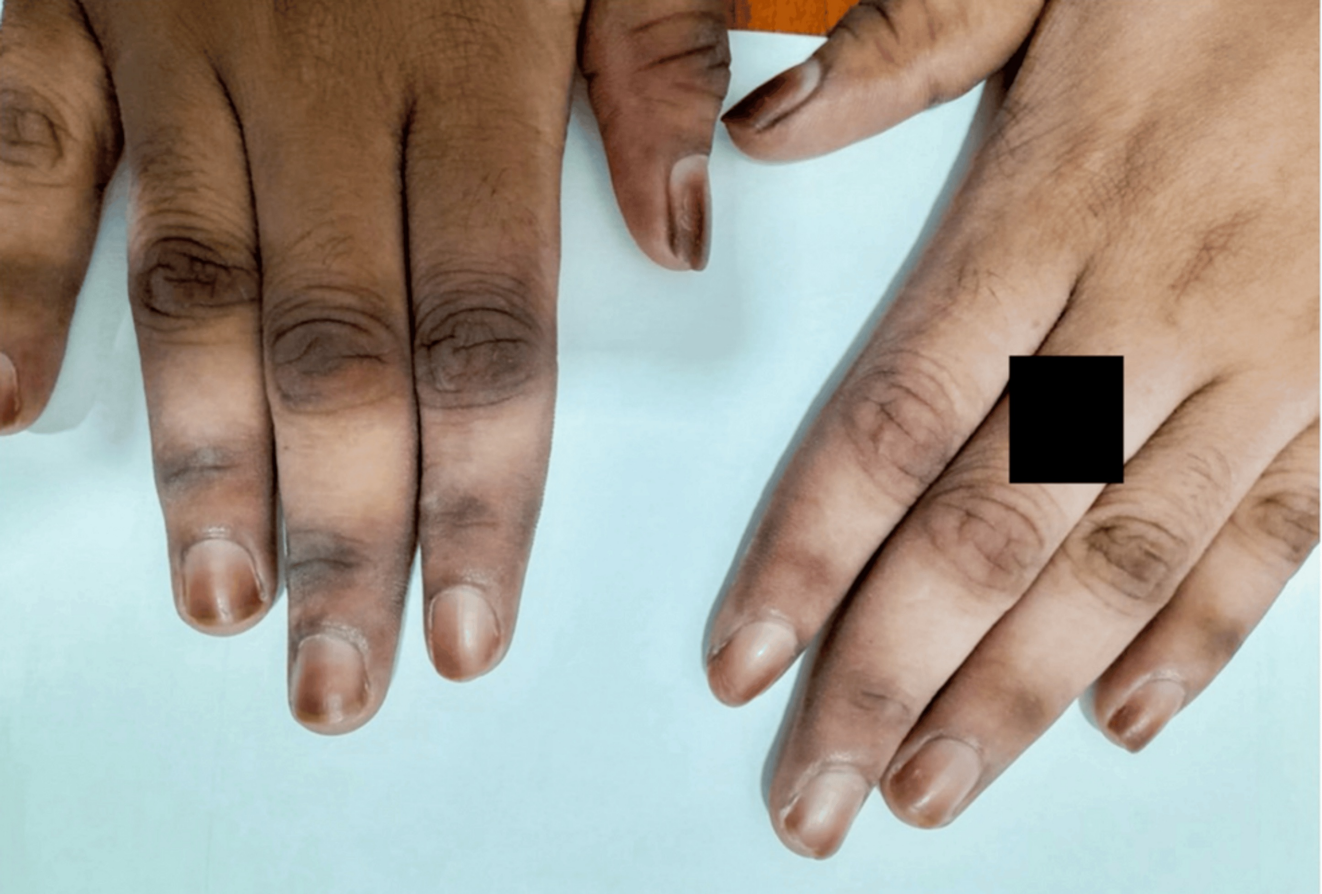
Clinical findings of Case 3

A 26-year-old female presented with dark brown hyperpigmentation involving the mid to distal nail plate of the fingernails in both hands, as shown in Figure 3. There was no alteration in nail shape or texture. She had previously been investigated for half-and-half nails, as seen in chronic kidney disease, but her metabolic profile was normal. Upon further inquiry, she had been using the OTC skin-depigmenting cream over her face and hands for nearly eight months intermittently with onset following four months of use. There was significant skin lightening on the dorsum of the hands, sparing the hyperpigmented areas on the interphalangeal joints, which retained their usual color.

Case 4

**Figure 4:**
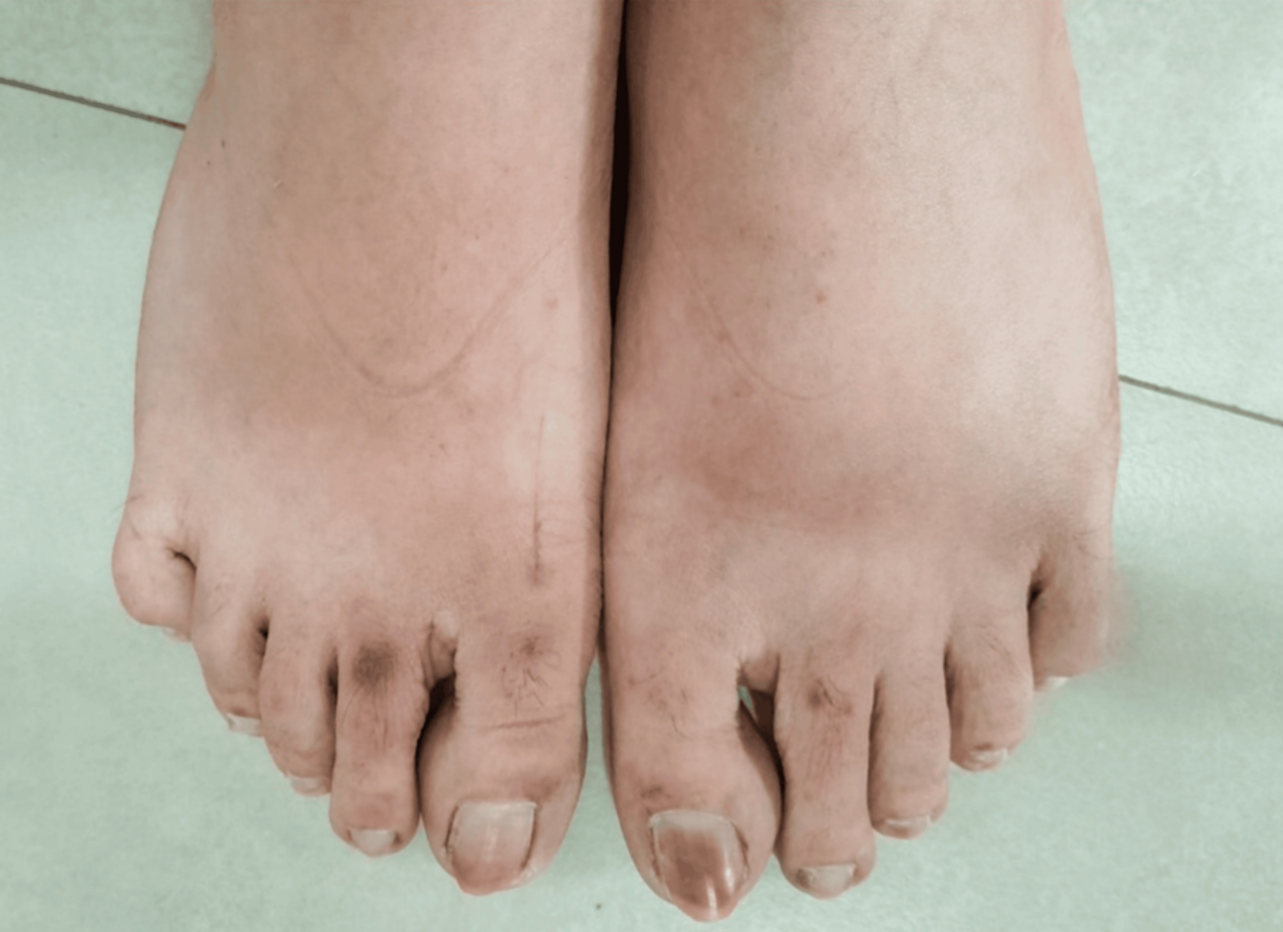
Clinical findings of Case 4

A 26-year-old female presented with light brown discoloration of the first toenail on both feet, more prominently on the left, as depicted in Figure 4. She had been applying the cream to her feet for approximately four months, and the hyperpigmentation occurred after three months of use. Additionally, she exhibited significant skin lightening on the dorsum of her feet, except for a hyperpigmented area over the dorsum of bilateral second metatarsophalangeal joints.

Case 5

**Figure 5:**
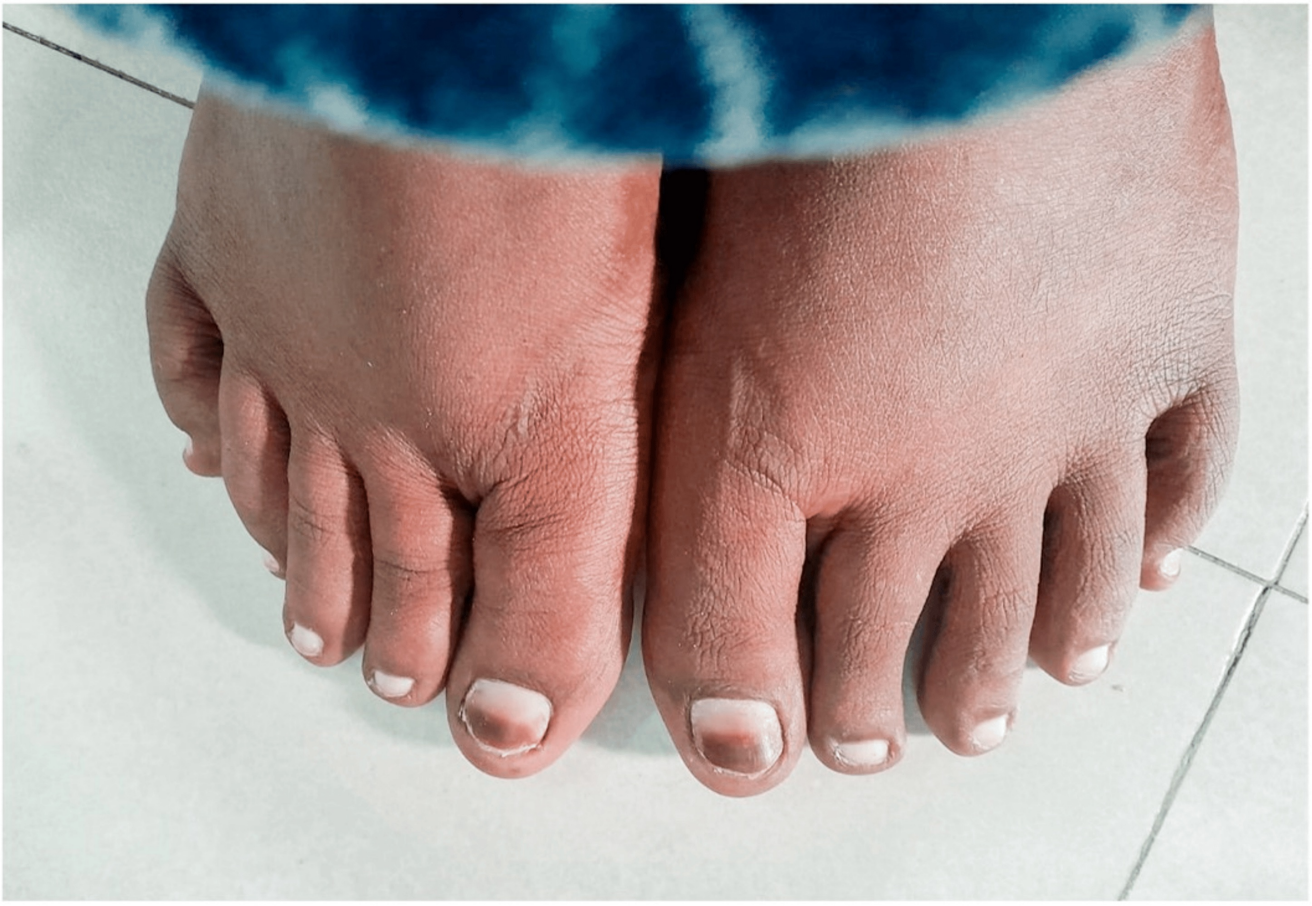
Clinical findings of Case 5

A 29-year-old female, with no prior co-morbidities, presented with significant nail hyperpigmentation of the first toenails on both feet, as shown in Figure 5. She had been applying the cream to the dorsal surface of her feet for nearly five months, with the findings appearing after 13 weeks of use. There were no changes in the shape or texture of the nails.

In all patients, no other mucosal or skin lesions were observed, and investigations revealed normal basic counts and metabolic profiles. The clinical findings of each patient are summarized in Table 1.

**Table 1:** Summary of clinical findings of the patients

Index	Gender	Age	Clinical Findings
Case 1	Female	25	Hyperpigmentation involving distal nail plate of bilateral hand fingernails
Case 2	Female	22	Hyperpigmentation of mid to distal nail plate of bilateral hand fingernails
Case 3	Female	26	Hyperpigmentation of mid to distal nail plate of bilateral hands
Case 4	Female	26	Hyperpigmentation of the first toenail of the left foot and slightly over the right foot
Case 5	Female	29	Hyperpigmentation of bilateral first toenails

All patients had used either skin-depigmenting creams on their bodies or faces for four to eight months. Three patients had used the same product (Cases 1, 2, and 4) and two patients (Cases 3 and 5) had used two different products. Sparing areas of the nail plate from hyperpigmentation correlated with cessation of the topical application of the whitening cream. Given the cultural context in Sri Lanka, where attire minimally exposes the skin, the patients applied the cream robustly to the face, hands, and, in some cases, the feet, explaining the toenail hyperpigmentation. The results of the presented patients were collected within a timeframe of 10 months.

## Discussion

Nail pigmentation can be caused by both internal and external factors, including nicotine, dyes, potassium permanganate, mercury compounds, HQ, elemental iron, mepacrine, photographic developer, anthralin, chrysarobin, glutaraldehyde, resorcin, as reported in the literature [6].

HQ has been known to be used in OTC skin-depigmenting products since the 1950s [2]. HQ inhibits tyrosine activity affecting the enzymatic oxidation of tyrosine to dopamine and its subsequent conversion to melanin. Further, by generating free radicals, it causes abnormal melanization and focal degradation of melanocytes leading to skin lightening [7]. HQ, a white compound, can be readily oxidized to a yellow compound, quinone, which further oxidizes to hydroxyquinone in a photosensitive reaction [8-10]. This unstable compound polymerizes upon further light exposure, forming dark brown products [8-10]. HQ or its breakdown products likely adhere to the nail surface rather than becoming incorporated into the nail plate. Garcia et al. mention that the color change results from HQ oxidation, and a similar color appears on the cream’s surface if exposed to air for long periods [11].

HQ is considered safe at a concentration of less or equal to 1% for cosmetic formulations designed for intermittent brief use followed by rinsing from the skin and hair, but not as leave-on beautifying products. The FDA proposed that HQ should not exceed 1.5-2.0% as an active ingredient in OTC skin-bleaching products [12]. Prolonged use of HQ is linked with various pigmentary adverse effects, including exogenous ochronosis and leukoderma with confetti-like depigmentation [2]. However, nail staining due to HQ has been reported in the literature in only a few cases [6,8-11,13-14].

Arndt and Fitzpatrick reported that three out of 56 patients in the United States who used HQ 2% or 5% for conditions such as melasma, vitiligo, nevus, freckling, and post-inflammatory hyperpigmentation, experienced nail pigmentation [13]. Garcia et al. noted diffuse orange and brown pigmentation of the nails after using skin-lightening cream containing 1% HQ [11]. However, the specific application area was not mentioned.

All the reported cases of nail pigmentation involved Caucasians from the UK and Australia [6,8-11,13-14]. Three women applied the cream to the back of their hands for senile and actinic lentigines, resolving within two to three months following discontinuation of the cream [8,9]. Another woman applied it to her trunk and neck, leading to hand nail pigmentation that resolved after she had used gloves before application [8]. Four males (including identical twin) experienced hand nail pigmentation after using 4% and 10% HQ cream for facial melasma, facial hyperpigmentation, and facial lentigines, with resolution within two to three months of stopping application [6,10,14], and recurrence one month after recommencement [10]. The hyperpigmentation in each case appeared as an asymptomatic darkening of the distal fingernails, affecting a varying number of nails on one or both hands [6,9,10,14]. The pigment was described as chestnut brown, with sun exposure leading to darkening and expansion of affected areas in some reports [8,9]. Asymmetrical nail involvement occurred depending on the finger used for application indicating local contact as the cause [6]. Despite the widespread use of HQ among Asiatic and Afro-Caribbean women for facial pigmentation, nail pigmentation appears underreported [9], possibly due to the masking of dyschromia by normal skin color [10].

Regarding the creams used by the patients in our case report, it is highly likely that they contained HQ, although, determining the exact concentration of HQ or the presence of other chemical constituents in each of these creams remains challenging. According to literature findings and patients' clinical presentations, nail hyperpigmentation appears to occur across a range of HQ concentrations, from 1% to 10%, with chronic use. We, therefore, recommend conducting further trials and studies involving a larger cohort of patients to evaluate these adverse effects at varying HQ concentrations, where permissible. Additionally, it is essential to investigate the long-term side effects in these patients.

## Conclusions

The presented patients, from Sri Lanka, had been using skin-lightening creams for four to eight months, developing asymptomatic nail pigmentation bilaterally in hands and, in some cases, feet, which resolved after discontinuation. This report features nail pigmentation associated with skin-lightening creams as observed in the Asiatic racial group, including the involvement of effects on toenails.

Therefore, patients presenting with nail pigmentation should be thoroughly assessed for potential exposures, including cosmetics, industrial exposure, and topical medications. HQ should be considered a potential cause of nail pigmentation given the growing trend of skin whitening. As mentioned in the given literature, gloves can be used to help mitigate the side effects of medically prescribed HQ. However, the unsupervised use of these creams, especially concerning dosage, duration, and extent of application, poses significant health risks. This underscores the need for more stringent oversight and control over the availability and usage of such creams. Patients, healthcare providers, and authorities must be aware of the potential adverse effects of non-licensed OTC whitening creams. Healthcare providers and dermatologists should recognize that pigmentation can be reversed by simply discontinuing exposure, preventing unnecessary investigations. Healthcare authorities should ensure that skin-lightening creams contain acceptable amounts of HQ, conduct post-marketing surveillance, and enhance regulation and public health awareness.
